# Cytosolic PhospholipaseA2 Inhibition with PLA-695 Radiosensitizes Tumors in Lung Cancer Animal Models

**DOI:** 10.1371/journal.pone.0069688

**Published:** 2013-07-19

**Authors:** Dinesh Thotala, Jeffrey M. Craft, Daniel J. Ferraro, Rama P. Kotipatruni, Sandeep R. Bhave, Jerry J. Jaboin, Dennis E. Hallahan

**Affiliations:** 1 Department of Radiation Oncology, Washington University in St. Louis, St. Louis, Missouri, United States of America; 2 Siteman Cancer Center, Washington University in St. Louis, St. Louis, Missouri, United States of America; 3 Hope Center, Washington University in St. Louis, St. Louis, Missouri, United States of America; 4 Mallinckrodt Institute of Radiology, Washington University in St. Louis, St. Louis, Missouri, United States of America; 5 School of Medicine, Washington University in St. Louis, St. Louis, Missouri, United States of America; Institut Gustave Roussy, France

## Abstract

Lung cancer remains the leading cause of cancer deaths in the United States and the rest of the world. The advent of molecularly directed therapies holds promise for improvement in therapeutic efficacy. Cytosolic phospholipase A2 (cPLA_2_) is associated with tumor progression and radioresistance in mouse tumor models. Utilizing the cPLA_2_ specific inhibitor PLA-695, we determined if cPLA2 inhibition radiosensitizes non small cell lung cancer (NSCLC) cells and tumors. Treatment with PLA-695 attenuated radiation induced increases of phospho-ERK and phospho-Akt in endothelial cells. NSCLC cells (LLC and A549) co-cultured with endothelial cells (bEND3 and HUVEC) and pre-treated with PLA-695 showed radiosensitization. PLA-695 in combination with irradiation (IR) significantly reduced migration and proliferation in endothelial cells (HUVEC & bEND3) and induced cell death and attenuated invasion by tumor cells (LLC &A549). In a heterotopic tumor model, the combination of PLA-695 and radiation delayed growth in both LLC and A549 tumors. LLC and A549 tumors treated with a combination of PLA-695 and radiation displayed reduced tumor vasculature. In a dorsal skin fold model of LLC tumors, inhibition of cPLA_2_ in combination with radiation led to enhanced destruction of tumor blood vessels. The anti-angiogenic effects of PLA-695 and its enhancement of the efficacy of radiotherapy in mouse models of NSCLC suggest that clinical trials for its capacity to improve radiotherapy outcomes are warranted.

## Introduction

Lung cancer is a leading cause of cancer death in United States. The American Cancer Society’s estimates in 2012 indicate that there were over 225,000 new cases and over 160,000 deaths from lung cancer in United States [1]. Radiation therapy (RT) remains an integral part of lung cancer management [2]. In the past decade, there have been substantial improvements in radiation treatment outcomes attributable to advances in clinical, physics, and biology research [3]. Despite these improvements in therapeutic regimens, local recurrence of lung cancer remains a persistent problem [4]. Most patients with unresectable non–small cell lung cancer (NSCLC) have a poor prognosis with median survivals of approximately 18 months, despite aggressive therapy [5], [6]. Thus, there is an urgent need to develop more effective approaches for the treatment of NSCLC.

Ionizing radiation (IR) not only damages nuclear DNA, but also activates a series of signaling cascades within the cell [7], [8]. Phospholipase A2 (PLA_2_, catalyzes the hydrolysis of membrane phospholipids at the SN-2 position to release lipid second messengers [9]. Ionizing radiation activates cytosolic phospholipase A2 (cPLA_2_) in endothelial cells [10]. After activation, cPLA_2_ cleaves palmitic acid to form phosphatidylcholine (PC) [11], [12], [13], [14] which then leads to production of lysophosphatidylcholine (LPC), lysophosphatidic acid (LPA), prostaglandin E_2_ (PGE2) and arachidonic acid [15], [16], [17], [18]. Arachidonic acid and LPA play important role in invasion and signaling during cancer progression [14], [19], [20], [21]. The activation of cPLA_2_ stimulates proliferation of endothelial cells and promotes the formation of vascular networks [16], [17]. LPC triggers the downstream activation of phosphatidylinositol 3-kinase (PI3K)/Akt and mitogen-activated protein kinase(MAP)/extracellular signal regulated kinase (ERK), which. results in increased cell viability of the endothelium in the tumor microenvironment [16], [17], [22]. Activation of cPLA_2_ in the tumor microenvironment leads to increased vasculature and enhanced tumorogenesis leading to radioresistance of the tumor and diminishing the efficacy of the radiotherapy [23], [24]. Combination of irradiation with inhibition of cPLA_2_ in preclinical lung cancer tumor models has been shown to suppressed tumor growth and reduced angiogenesis [25], [26].

Previous investigations have used cPLA_2_ inhibitors unsuited for translation to the clinic due to their toxicity [17]. We studied the effects of PLA-695, a cPLA_2_ inhibitor that has already been tested in clinical trials. The phase I study (NCT00366262) evaluating the safety of PLA-695 compared to placebo and naproxen has been completed (clinical trials.gov). Later, the Phase II clinical trial (NCT00396955) compared 4 dose regimens of PLA-695, naproxen, and placebo in subjects with osteoarthritis of the knee (clinical trials.gov). The present study determined the efficacy of PLA-695 in combination with irradiation to treat mouse models of lung cancer.

We found that PLA-695 inhibits radiation induced phosphorylation of ERK and Akt in cultured endothelial cells. PLA-695 in combination with irradiation prevented endothelial cell migration. PLA-695 enhanced radiation induced cell death and attenuated invasion of lung cancer cells. PLA-695 inhibited the formation of new blood vessels and angiogenesis [26]. In addition, PLA-695 enhanced the efficacy of radiation in two mouse models of lung cancer.

## Methods

### Cell Culture and Treatment

Primary culture of Human Umbilical Vein Endothelial Cells (HUVECs) pooled from multiple donors was obtained from Cambrex (East Rutherford, NJ, USA) and maintained in complete EBM-2 medium (Cambrex). Cells from passages 2–5 were used in this study. HUVECs were starved for 1 hour before treatment in additive-free EBM-2 medium. 3B11 microvascular cells were obtained from American Type Culture Collection (Manassas, VA) and maintained in DMEM with 5% fetal bovine serum (FBS). Cells from passages 3–6 were used in this study. 3B11s were starved in DMEM +1% FBS for 3 hours prior to all studies. PLA-695 was obtained from Pfizer Inc under the Pfizer-WU biomedical agreement. For the irradiation of cells, Therapax 250 X-ray machine (Pantak Inc., East Haven, CT, USA) delivering 2.04 Gy/min at 250 kVp was used. Due to high sensitivity of HUVECs to temperature and pH, cells were maintained in an incubator adjacent to the irradiator prior to and after treatment. In experiments with PLA-695, cells were treated for 45 minutes prior to IR (3 Gy) with either dimethylsulfoxide (DMSO, vehicle control) or varying concentration of drug in DMSO (10–600 nM).

### Co-culture Clonogenic Survival Assay

HUVEC (1.0×10^6^) and bEnd.3 cells (1.0×10^6^) were plated in 100 mm plates and after 24 h, A549 (2×10^6^) and LLC (2×10^6^) cells were plated onto transwell inserts (Corning Inc., Corning, NY). After co-culture for 24 h, cells were treated with 300 nM of PLA-695 or vehicle control DMSO for 45 minutes prior to IR with 0, 2, 4, 6 or 8 Gy. After the treatments as co-culture with either PLA-695 or DMSO calculated numbers of LLC and A549 cells were plated to enable normalization for plating efficiencies. Clonogenic assays were also performed with LLC and AF549 alone. Fixed numbers of cells were plated to enable normalization for plating efficiencies. Cells were allowed to attach for 5 hours and then treated with 300 nM of PLA-695 or vehicle control DMSO for 45 minutes prior to IR with 0, 2, 4, 6 or 8 Gy. After 7–10-day incubation plates were fixed with 70% EtOH and stained with 1% methylene blue. Colonies consisting of >50 cells were counted by viewing the plates under a microscope. The survival fractions were calculated as (number of colonies/number of cells plated)/(number of colonies for corresponding control/number of cells plated).

### Colorimetric Cell Proliferation Assay

Cell proliferation was determined using cell titer 96 Aqueous Non-Radioactive Cell Proliferation Assay reagent (Promega). The assay was performed following the manufacturers protocol. Briefly cells were treated with either DMSO control or 300 nM PLA-695 for 45 minutes and irradiated with 3 Gy. The medium was changed after 1 hour and the plates were incubated for 96 h. Cell viability was measured colorimetrically after 96 h by measuring absorbance at 490 nm. Experiments were performed in triplicate and standard errors were calculated.

### Morphologic Analysis of Cells Stained with DAPI

LLC and A549 cells were grown in chamber slides and treated with either DMSO or 300 nM PLA-695 for 45 minutes and then irradiated with 3 Gy. At 96 h post-irradiation, cells were fixed in 4 paraformaldehyde and then stained with 2.5 µgml 4′,6-diamidino-2-phenylindole (DAPI) in phosphate-buffered saline. Photomicrographs were taken using an Olympus BX60 fluorescent microscope equipped with digital camera. Multinucleated cells and cells containing giant nuclei were counted in several randomly selected fields. The average percentage of such cells over total cell number was calculated from three experiments.

### Tumor Transwell Invasion Assay

The tumor Transwell Matrigel Invasion Assay was used to monitor tumor-endothelium interactions and cell migration. This assay employs a simplified Boyden chamber-like design that consists of two chambers separated by a filter coated with Matrigel. A549 (1.0×10^6^ cells/well) or LLC (0.6×10^6^ cells/well) were suspended in serum-free media and added to the top of 24 well plates with 8 µm basement membrane matrix-coated polycarbonate membrane inserts (Bedford, MA, USA). 500 uL of fresh medium was added to the bottom chamber as chemo-attractant. For radiosensitization studies, both chambers were then treated with vehicle DMSO or 300 nM PLA-695 for 45 minutes prior to IR with 4 Gy. Cells migrated from the top chamber through the coated filter pores to the bottom of the filter. After 24 hours, remaining cells in the upper chamber of the membrane inserts were removed using a wet cotton swab. The cells that adhered on the outer surface of the transwell insert membrane which had invaded through the Matrigel were fixed with 100% methanol and stained. Cells that had invaded in 7–10 high power field (HPF) from each sample were counted using Image J Software (NIH, Bethesda, MD), and the average number of cells that invaded through the membrane per HPF was calculated. Mean and standard error for each treatment group were calculated for each group.

### Signal Transduction Pathway Analysis

After treatment, HUVECs cells were harvested at the indicated times. Total protein extraction was performed using M-PER reagent (Pierce, Rockford, IL, USA) containing protease inhibitors and phosphatase inhibitors II and III (Sigma, MO). Protein concentration was quantified using BCA Reagent (Pierce). Protein extracts (40 µg) were subjected to western immunoblot analysis using antibodies for the detection of phospho-Akt (Thr308/Ser473), phospho-ERK1/2, (Thr202/Tyr204), total Akt, and total ERK1/2 (all from Cell Signaling Technologies.

Danvers, MA, USA). Antibody to actin (Sigma-Aldrich, St. Louis, MO) was used to evaluate protein loading in each lane. Immunoblots were developed using the Western Lightning Chemiluminescence Plus detection system (PerkinElmer, Wellesley, MA, USA) according to the manufacturer’s protocol.

### Cell Migration

HUVEC and bEND3 cells were grown to 70%–80% confluency in 60 mm plates. Three parallel wounds were created on each plate by scratching the cell monolayer with a 200-µL pipette tip, and boundaries of the wounds were marked. Cells were treated for 1 hour with vehicle (DMSO) or 300 nM PLA-695, followed by 3 Gy IR. After 24 hours, the cells were photographed, and cells found inside and outside of the wound boundaries were counted from six HPFs per sample. Cells that spanned the boundaries were classified as non-migrated cells. Cell density within the wound is presented as a percentage of total cell density in the plate. Results are from triplicate samples in three independent scratch assays.

### Tumor Growth Delay

The Institutional Animal Care and Use Committee of Washington University in St. Louis specifically approved this study. LLC (10^6^) or A549 (10^6^) cells were implanted into the right footpad C57/BL6 mice. Once all tumors became measurable by plethysomography, as determined by volume of tumor-bearing footpad minus volume of contralateral footpad, the mice were serpentine sorted into groups of six to seven animals representing similar distributions of tumor sizes (range = 40–70 mm^3^). Tumor–bearing mice were injected intraperitoneally with vehicle (DMSO) or PLA-695 at 7.5 mg per kg body weight once daily for five consecutive days. Thirty minutes after drug injection mice were anesthetized with isoflurane and positioned in the Pantak irradiator and irradiated with 2 Gy daily for five consecutive days for a total of 10 Gy. Lead blocks (10 mm thick) were used to shield the head, thorax, and abdomen. Tumor size was monitored longitudinally with plethysmography. Mice were sacrificed by CO^2^ asphyxiation once tumors reached a volume of approximately 700 mm^3^ or when ulceration became apparent on the footpad per Animal Care guidelines.

### In vivo Angiogenesis Assay Dorsal Skin-fold Chamber Model

The implantation technique of the dorsal skin-fold chamber model has been described previously [27]. Briefly, diffusion chambers containing LLC cells (1×10^6^ cells per chamber) were inserted in the dorsal air sac made by making a superficial incision horizontally along the edge of the dorsal air sac. The skin was carefully sutured after placing the chambers underneath the mice skin. The mice treatments were performed 5 to 7 days following surgical insertion of the diffusion chambers. The skin fold covering the chambers was carefully removed after euthanizing the mice and photographed under visible light. The number of tumor induced blood vessels was counted in ten different fields within the chamber in the area of the air sac fascia.

### Histological Analysis of Tumor Vasculature

The tumor bearing mice were sacrificed 24 hours after the final treatment and the tumors were resected, fixed in formalin, embedded in paraffin and sectioned. Sections (5 µm thick) were treated with 20 µg/ml proteinase K for 30 minutes at room temperature and then were incubated overnight with a rabbit polyclonal antibody against human Von Willebrand factor (vWF) (1∶100 dilution; Dako, Carpinteria, CA). Tissue sections were subsequently incubated with Alexa Fluor 488–conjugated goat anti-rabbit IgG (1∶500 dilution; Invitrogen Molecular Probes) for 1 hour at room temperature. Alexa Fluor 488–stained vessels were counted in three randomly selected HPFs on each of three sections per tumor. Vascularity was determined as the average number of stained vessels per HPF. Tumor vascular cross sectional area was determined similarly, using NIH Image J software, in which the area enclosed by vessels was divided by total area of the HPF to determine percent vascular area.

### Statistical Analysis

The mean and standard error of the mean (SEM) of each treatment group were calculated for all experiments. The number of samples is indicated in the description of each experiment. Students T-test was use to compare two treatment groups. Analysis of variance (ANOVA) was used to compare treatment effectiveness in tumor growth delay studies. A *p-*value of <0.05 was considered statistically significant.

## Results

### Effect of PLA-695 on Pro-survival Signaling after Radiation

**Figure 1:**
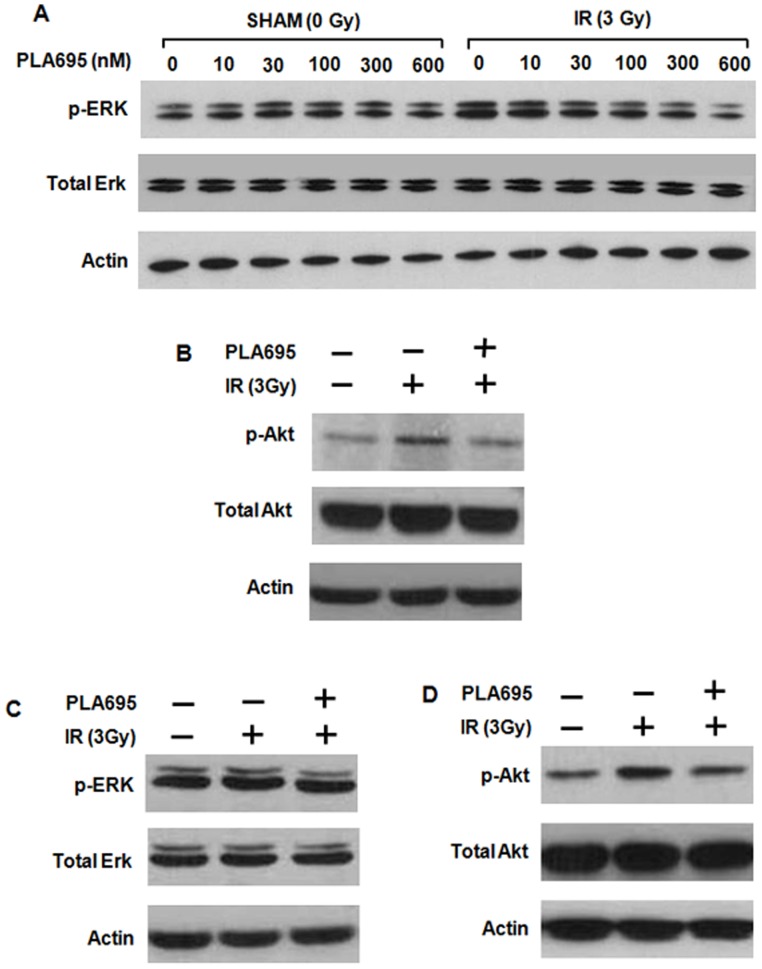
Effect of PLA-695 on pro-survival signaling after radiation. HUVEC cells (A) were treated with various concentrations of PLA-695 as indicated for 45 min before treatment with 3 Gy, cells were lysed at 5 min after IR. HUVEC (B) and 3B11 (C&D) cells were treated with 300 nM PLA-695 for 45 min before treatment with 3 Gy. Cells were lysed at 5 min after IR. Shown are immunoblot analyses using specific antibodies to phospho-Akt^Ser473^, total Akt, phospho-ERK1/2, total ERK1/2, and actin.

Activation of ERK and Akt occurs rapidly following radiation in HUVEC cells (Fig. 1). Treatment with PLA-695 attenuates the IR induced ERK phosphorylation in a dose-dependent manner (Fig. 1 A). ERK activation is reduced to baseline (sham-treated cells) by 300 nM of PLA-695. At higher concentrations, (600 nM), the levels of ERK phosphorylation fall below baseline levels. The IR induced Akt phosphorylation was also abrogated when HUVEC cells were treated with 300 nM of PLA-695(Fig. 1 B). We found similar results in 3B11 cells treated with 300 nM PLA-695(Fig. 1 C and D).

### PLA-695 Sensitizes A549 and LLC Cells to Ionizing Radiation

**Figure 2:**
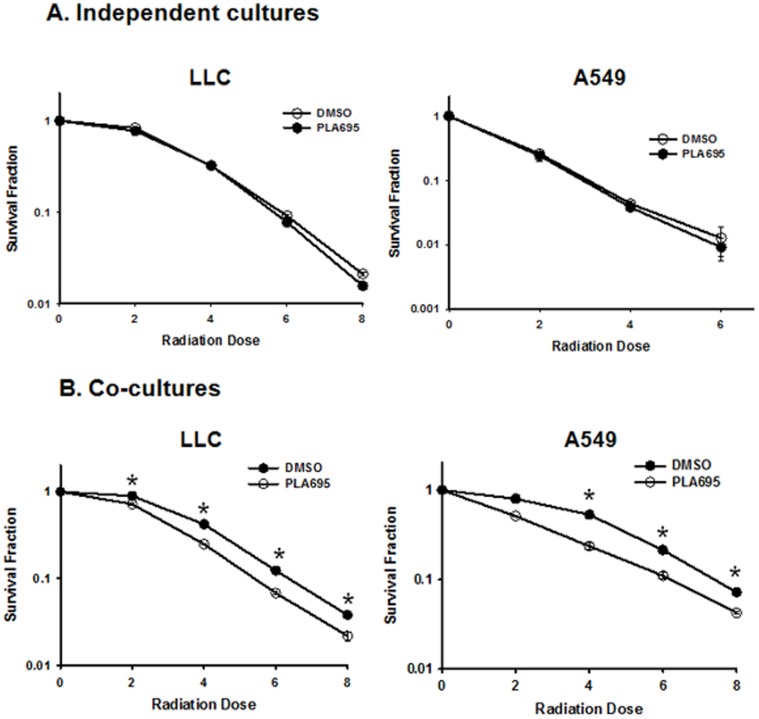
PLA-695 enhances clonogenic cell death in LLC and A549. LLC and A549 cells were grown independently (A) or as co-cultures (B) with bEND3 and HUVEC cells and were treated with DMSO or 300 nM PLA-695 in serum free media for 45 min prior to IR. The cells were then irradiated with 0, 2, 4, 6 and 8 Gy and plated for clonogenic survival assay. After 7–10 days, cells were stained with 1% methylene blue and colonies consisting of >50 cells were counted by microscopy. Surviving colonies were normalized for plating efficiency. Shown are average survival fractions and SEM.

To evaluate the role of cPLA_2_ in the viability of irradiated lung cancer cells, we treated mouse lung cancer cell line LLC and human lung cancer cell line A549 with 300 nM of PLA-695 for 45 minutes prior to irradiation. We performed clonogenic assays either as independent cultures (Fig. 2A) or in co-culture (Fig. 2B) with endothelial cells (LLC with bEND3 and A549 with HUVEC). The plating efficiency of LLC cells was 66% for DMSO treated cells and 60% for PLA-695 treated cells. The plating efficiency of A549 cells was 64% for DMSO treated cells and 52% for PLA-695 treated cells. The cells were normalized for plating efficiency when calculating the survival fraction for clonogenic assays. Pretreatment of lung cancer cell lines with PLA-695 prior to IR had no impact on clonogencity when grown as independent cultures of either in LLC (2 Gy P = 0.53, 4 Gy P = 0.87, 6 Gy P = 0.02, 8 Gy P = 0.01) or A549 (2 Gy P = 0.753, 4 Gy P = 0.441, 6 Gy P = 0.636) (Fig. 2A). LLC and A549 cells grown as co-cultures and treated with PLA-695 prior to IR showed a significantly decreased cell survival in both LLC cells (2 Gy P = 0.003, 4 Gy P<0.001, 6 Gy P<0.001, 8 Gy P = 0.012) and A549 cells (2 Gy P = 0.163, 4 Gy P<0.001, 6 Gy P<0.001, 8 Gy P = 0.031) as compared to cells treated with radiation alone (Fig. 2B). Dose enhancement factors were calculated at 10% cell survival by dividing the dose of radiation from the radiation-only survival curve with the corresponding dose from the PLA-695 plus radiation curve. The dose enhancement factors were 1.17 for the LLC cells, 1.28 for A549 cells (Fig. 2B).

### PLA-695 Reduces Proliferation in Endothelial and Lung Cancer Cells after Irradiation

**Figure 3:**
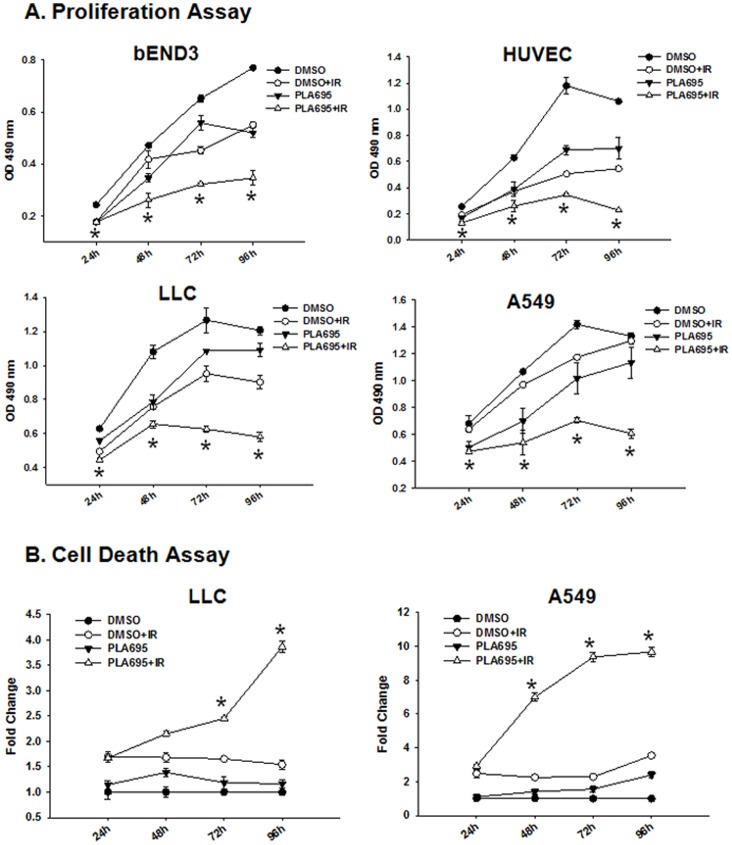
A. PLA-695 reduces proliferation in endothelial and lung cancer cells after irradiation. Equal numbers of bEND3, HUVEC, LLC and A549 cells were plated in 96 well plates and treated with 300 nM PLA-695 for 45 min prior to treatment with 3 Gy. Cell proliferation was determined using a colorimetric cell proliferation assay at 24, 48, 72 and 96 h post treatment. Shown is the absorbance at 490 nm. **B. PLA-695 enhances cell death in irradiated lung cancer cells**. LLC and A549 cells were treated with 300 nM PLA-695 or DMSO for 45 min prior to treatment with 3 Gy. Cells were stained with Annexin V-APC and propidium iodide and analyzed by flow cytometry at 24, 48, 72 or 96 h after irradiation. Shown are the line graphs indicating fold increase of cell death over control for each treatment with SEM from three experiments.

To elucidate the role of cPLA_2_ in both the endothelial cells and lung cancer cells, we investigated how PLA-695 affects proliferation in LLC, A549, bEND3 and HUVEC cells (Fig. 3A). Equal numbers of LLC, A549, bEND3 and HUVEC cells were plated in a 96 well plate. The following day, cells were treated with 300 nM of PLA-695 for 45 minutes prior to IR. The plates were read at 24, 48, 72, or 96 h using the colorimetric cell proliferation assay by measuring the absorbance at 490 nm. bEND3 cells treated with PLA-695 alone showed a significant reduction in cell proliferation compared to DMSO at 24 h (P <0.001), 48 h (P = 0.002) and 96 h (P <0.001) and at 72 h (P = 0.047). bEND3 cells treated with combination treatment of PLA-695 with IR showed a significant reduction in cell proliferation compared to IR alone at 72 h (P = 0.001) and 96 h (P = 0.002) and reduced proliferation at 24 h (P = 1.0) and 48 h (P = 0.023). HUVEC cells treated with PLA-695 alone showed a significant reduction in cell proliferation compared to DMSO at 24 h (P = 0.003), 48 h (P = 0.016), 72 h (P = 0.013), and 96 h (P <0.001). HUVEC cells treated with combination treatment of PLA-695 with IR showed a significant reduction in cell proliferation compared to IR alone at 24 h (P = 0.004) 72 h (P = 0.002) and 96 h (P <0.001). LLC cells treated with PLA-695 alone showed reduction in cell proliferation compared to DMSO at 24 h (P = 0.076), 48 h (P = 0.017), 72 h (P = 0.028) and 96 h (P = 0.175). LLC cells treated with combination treatment of PLA-695 with IR showed a significant reduction in cell proliferation compared to IR alone at 24 h (P = 0.001), 48 h (P = 0.010), 72 h (P <0.001) and 96 h (P <0.001). A549 cells treated with PLA-695 alone showed a reduction in cell proliferation compared to DMSO at 24 h (P = 0.017), 48 h (P = 0.008), 72 h (P = 0.070) and 96 h (P = 0.079). A549 cells treated with a combination treatment of PLA-695 with IR showed significant reduction in cell proliferation compared to IR alone at 72 h (P = 0.003) and 96 h (P = 0.003).

### PLA-695 Enhances Cell Death in Irradiated Lung Cancer Cells

cPLA_2_ has been shown to activate signaling through the Ras/Raf/ERK pathway [10], [28]. Activation of phospholipase A2 has also been indicated to be the cause of delayed cell death induced by H_2_O_2_ [29]. We studied cell death in LLC and A549 by staining for Annexin V and,PI using flow cytometry at 24, 48, 72 and 96 h after IR (Fig. 3B). PLA-695 treatment of LLC cells did not induce any significant cell death at all of the tested time points. A549 cells treated with PLA-695 showed a slight increase in cell death with time. IR alone induced a level of cell death in both LLC and A549 cells that was similar at all the time points tested (24, 48, 72 and 96 h). Combined treatment with PLA-695 and IR did not induce increased cell death compared to IR alone at 24 h in both LLC cells (P = 0.831) and A549 cells (P = 0.192). At 48 hours the combined treatment showed modest cell death in LLC cells (P = 0.100) and significant cell death in A549 cells (P <0.001). At 72 h and 96 h, the combined treatment showed significant cell death in both LLC cells (P <0.001) and A549 cells (P <0.001).

**Figure 4:**
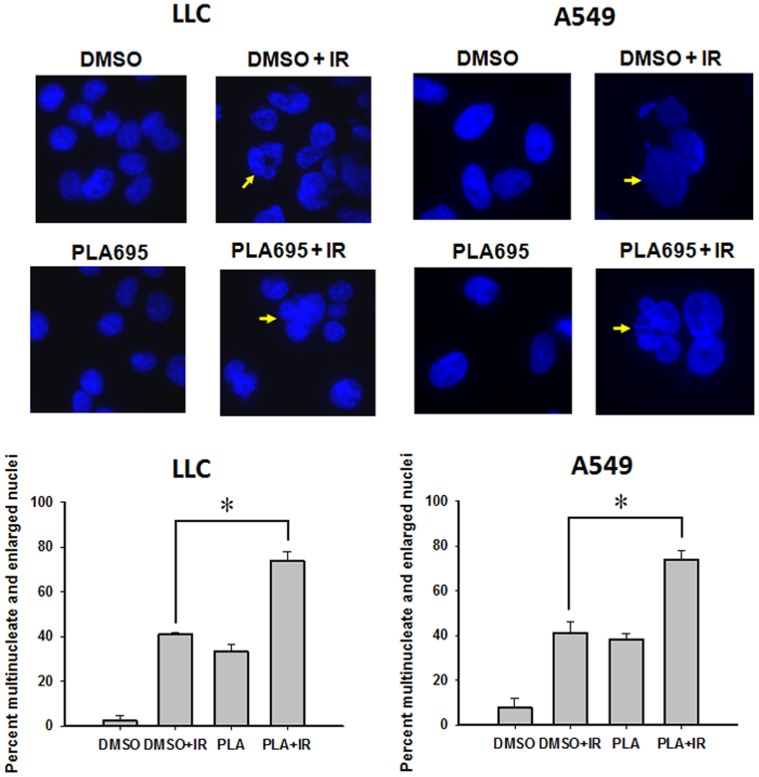
PLA-695 enhances cell death in irradiated lung cancer cells. LLC and A549 cells were grown in chamber slides were treated with 300 nM PLA-695 or DMSO for 45 min prior to irradiation with 3 Gy. Cells were fixed 96 h later and stained with 4′,6-diamidino-2-phenyllindole (DAPI). Shown are the micrographs of DAPI stained cells, arrows indicate multinucleate cells and giant cells. Multinucleated and giant cells were counted in five randomly selected fields. A bar graph depicting the average percent of multinucleate/giant cells for each treatment is shown.

We next evaluated the nuclear morphology of irradiated cells using DAPI staining (Fig. 4); the results were similar to the assessment done by Annexin V/propidium iodide staining**.** At 96 h post irradiation, the combined treatment with PLA-695 and IR resulted in significantly more multinucleate cells and cells with enlarged nuclei (characteristic of mitotic catastrophe) in both LLC cells and A549 cells (P 0.006), when compared to IR alone (LLC, P = 0.001; A549, P = 0.006) and PLA695 alone(LLC, P = 0.001; A549, P = 0.002).

### PLA-695 Reduces Endothelial Cell Migration after Radiation

**Figure 5:**
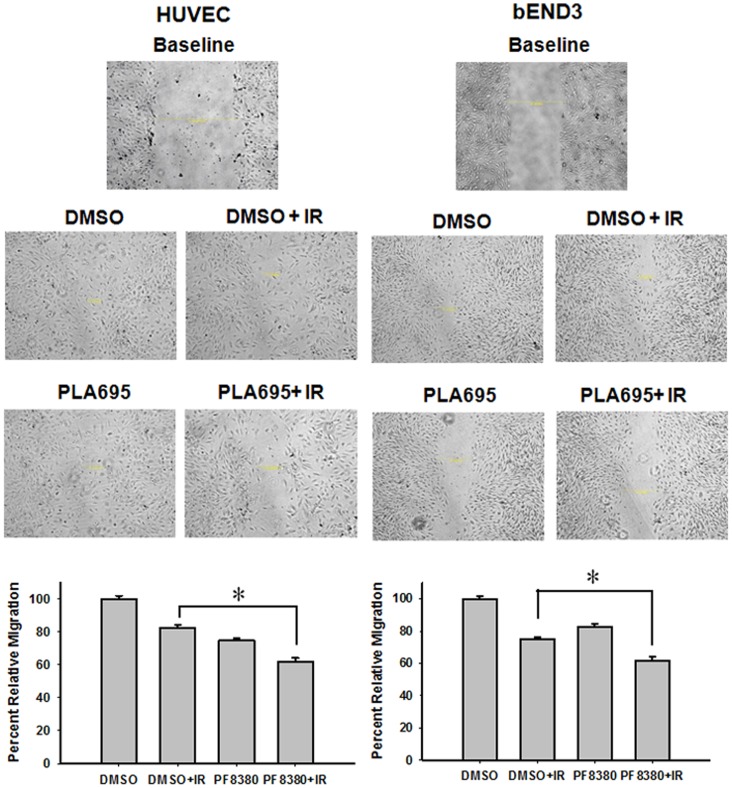
PLA-695 reduces endothelial cell migration after radiation. HUVEC and bEND3 cells were plated on 60 mm plates and allowed to grow to 80% confluency. The semi-confluent cell layer was scraped using a 200 µl sterile pipette tip to create a scratch/wound devoid of cells. The remaining cells were treated with vehicle control or 300 nM PLA-695 for 45 min prior to IR with 3 Gy. Migration was assessed at 24 h after treatment. The number of cells migrated in the scratch/wound were counted and normalized to surrounding cell density per HPF. Shown are representative photomicrographs and bar graphs representing the mean percentages of migrating cells relative to corresponding controls with SEM from three experiments.

We performed gash closure assays with HUVEC and bEND3 cells to determine the effect of PLA-695 (Fig. 5). In this assay, a wound was created in the cell culture(Baseline) and cells were treated with vehicle (DMSO), vehicle and 3 Gy (DMSO+IR), PLA-695 (300 nM), or a combination of PLA-695 and 3 Gy (PLA-695+IR). Cell migration was calculated by counting the number of cells moving into the wound (Fig. 5). We found that 24 hours after treatment, the gash was nearly completely covered in HUVEC and bEND3 cultures treated with DMSO. Irradiation alone led to a reduction in migration in HUVEC (82%) and bEND3 (77%) cells. Similar reduction was observed in cells treated with PLA-695 alone in both HUVEC (74%) and bEND3 (62%). The combination of PLA-695 and IR led to a significant reduction in the migration of HUVEC (61%; P = 0.003) and bEND3 (48%; P = 0.012) cells when compared to IR alone (Fig. 5).

### PLA-695 Reduces Tumor Cell Invasion after Radiation

**Figure 6:**
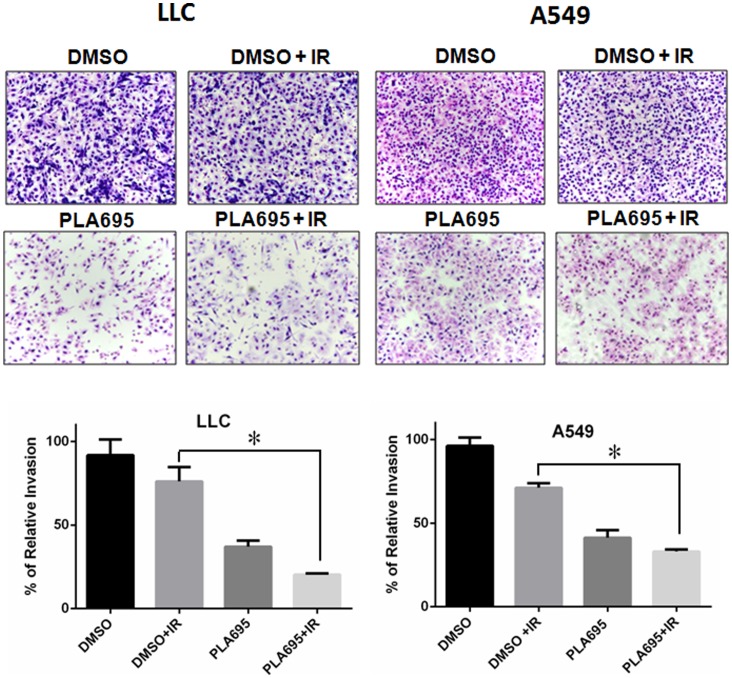
PLA-695 reduces tumor cell invasion after radiation. LLC and A549 cells were added to the 8 micron inserts and were treated with 300 nM PLA-695 or DMSO for 45 minutes prior to 3 Gy irradiation. Cells were allowed to invade/migrate from the top chamber through the coated filter pores to the complete medium at the bottom of the inserts for 48 hour. Cells were then fixed, stained and cells that invaded through the membrane was calculated by counting the number of cells per HPF. Shown are representative photomicrographs and a bar graph representing the number of invasive cells with SEM; * p>0.05.

cPLA_2_ has been implicated in cell migration, tubule formation [26] and invasion [30]. We examined the effect of PLA-695 on tumor cell invasion in LLC and A549 cell lines using the transwell-invasion assays. In LLC, we observed a 24% decrease in cell invasion when cells were irradiated with 4 Gy; however, pre-treatment with 300 nM PLA-695 prior to IR resulted in a 56% further reduction in cell invasion (P = 0.003; Fig. 6). Treatment of LLC cells with PLA-695 alone showed a 63% reduction in invasion compared to control. Similarly in A549 cells, radiation alone caused a 30% reduction in invasion, while pre-treatment with 300 nM PLA-695 prior to IR reduced invasion further by 37% (P <0.001; Fig. 6).Treatment of A549 cells with PLA-695 alone produced a 58% reduction in invasion compared to control.

### PLA-695 Radiosensitizes Tumors in Animal Models of NSCLC

**Figure 7:**
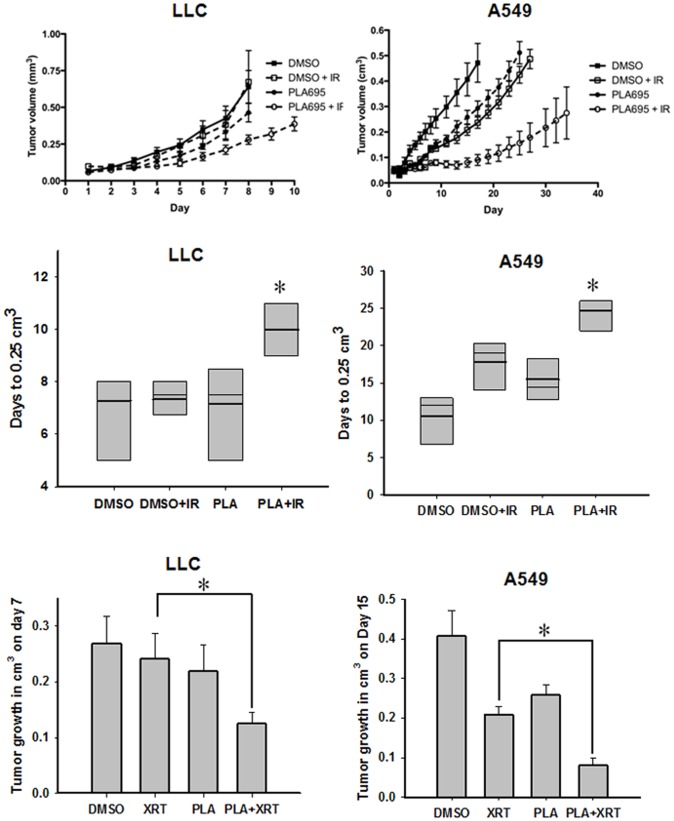
PLA-695 enhances tumor growth delay in heterotopic lung cancer tumors. LLC or A549 cells were implanted into the right footpad of C57/BL6 mice. Tumors were irradiated with 2 Gy for 5 consecutive days for a total of 10 Gy. Mice were treated with 7.5 mg/Kg body weight or vehicle control for 30 min prior to IR on days 1, 3, 5, 7 and 9. Shown are mean tumor volumes for LLC and A549 with SEM from each treatment group of mice. Tumor growth delay for LLC and A549 tumors was calculated as the number of days for tumors to reach 0. 25 cm^3^(B). Shown is a bar graph representing the mean tumor growth delay with SEM from each treatment group of 7 mice; * p<0.05. Tumor volumes were analyzed on day 7 for LLC tumors and day 15 for A549 tumors. Shown is a bar graph representing the tumor growth on day 7 for LLC tumors and day 15 for A549 tumors with SEM from each treatment group of 6 mice; * p<0.05.

To determine the efficacy of PLA-695 as a radiosesitizer in NSCLC, we used heterotopic LLC and A549 tumor models grown in C57 and nude mice, respectively. When the LLC and A549 tumors were palpable (range = 40–70 mm^3^), the mice were treated for five consecutive days with DMSO (vehicle), irradiation (IR) alone (10 Gy total, 5 fractions of 2 Gy), PLA-695 alone (7.5 mg/kg/day, intraperitoneal), or a combination of PLA-695 and IR. Subsequent tumor growth was monitored using a plethysmometer. We analyzed tumor growth delay in these experiments in two ways. First, the time for the tumor volume to reach a size of 0.25 cm^3^ was determined. Second, we analyzed the tumor volumes on day 7 for LLC tumors and day 15 for A549 tumors (Fig. 7). The LLC tumors grow much faster than A549 tumors. Untreated tumors and IR treated tumors took 7 days to reach a tumor volume of 0.25 cm^3^, while PLA-695 treated tumors took 7.5 days. Tumors treated with a combination of PLA-695 and IR took 9.5 days to reach a volume of 0.25 cm^3^. On day 7 the tumors treated with a combination of PLA-695 and IR were smaller (0.13 cm^3^) compared to untreated (0.27 cm^3^; P = 0.019), IR alone (0.24 cm^3^; P = 0.038) and PLA-695 alone (0.22 cm^3^; P = 0.100).

The enhancement of tumor growth delay by PLA-695 was more pronounced in A549 tumor-bearing nude mice (Fig. 7). The untreated A549 tumors reached the test volume (0.25 cm^3^) in 9 days, PLA-695 treated tumors (15 days) IR treated tumors (18 days) and PLA-695 in combination with IR treated tumors (32 days). On day 15, the A549 tumors treated with a combination of PLA-695 and IR were significantly smaller (0.08 cm^3^) than untreated tumors (0.407 cm^3^; P <0.001), or IR (0.208 cm^3^; P <0.001) or PLA-695 (0.258 cm^3^; P <0.001) treated tumors. Interestingly, two of the six tumors in the combination therapy arm did not grow further throughout the duration of the study (37 days after first treatment) and one tumor regressed completely.

### PLA-695 Damages Vessels in Tumors Treated with Radiation

**Figure 8:**
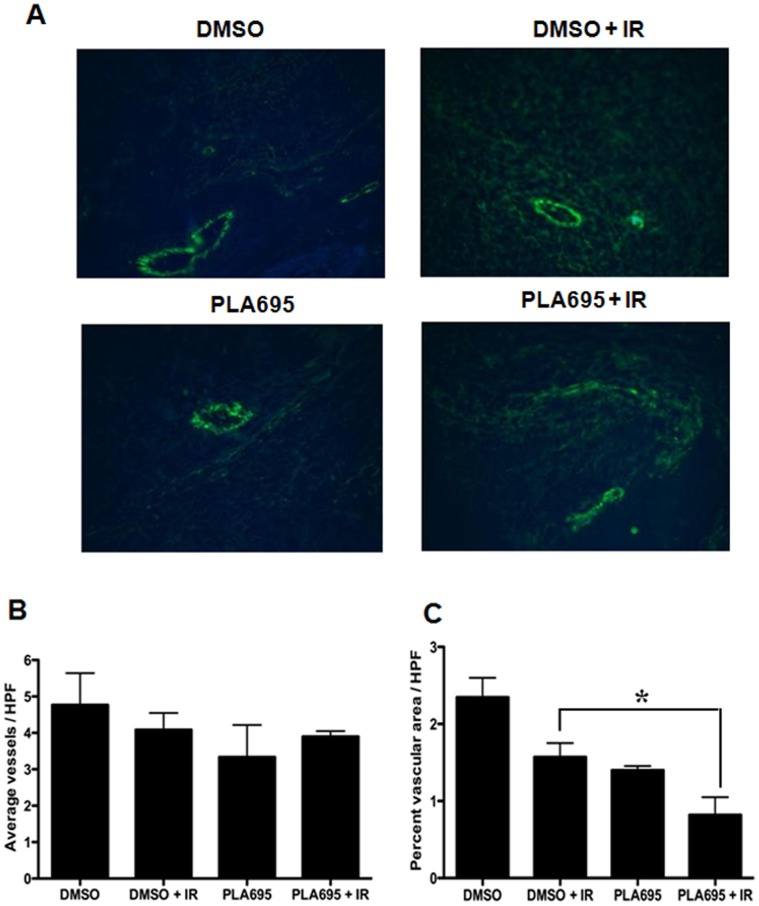
PLA-695 damages vessels in tumors treated with radiation. C57/BL6 mice with LLC tumors received i.p. injections of vehicle or 7.5 mg/kg PLA-695 30 min prior to IR with 2 Gy. Treatment was repeated for 5 consecutive days. Twenty-four hours after the final treatment, tumors were harvested, fixed in 10% formalin, sectioned into 5-µm sections and stained with anti-vWF antibody. Shown are representative micrographs of vWF-stained vessels and bar graphs of the average number of stained vessels per HPF and percent vascular area per HPF with SE from group of 5 mice; *, *P*<0.05.

To determine the effects of cPLA_2_ inhibition with PLA-695 on tumor vascularity, LLC tumors were treated similarly to those in the tumor growth delay study. Microvessel density and cross sectional area were determined by staining with an antibody against Von Willebrand factor, an endothelial cell marker (Fig. 8A). Analysis of sections of tumors subjected to various experimental treatments indicated that there was no significant difference in the number of vessels per high power field (HPF) in the various experimental groups (Fig. 8 B). Tumors treated with DMSO alone, PLA-695 alone and DMSO with IR had normal appearing microvasculature, while tumors treated with a combination of PLA-695 and IR had collapsed microvasculature (Fig. 8C). Quantitation of the vascular cross-sectional area per HPF in the treatments showed that there was a significant reduction in area with combination therapy of PLA-695 and IR (Fig. 8 C, p<0.05), while treatment with either radiation or drug alone showed no statistically significant reductions in vascular area.

### Combination Treatment of PLA-695 with Irradiation Decreases Vascularity

**Figure 9:**
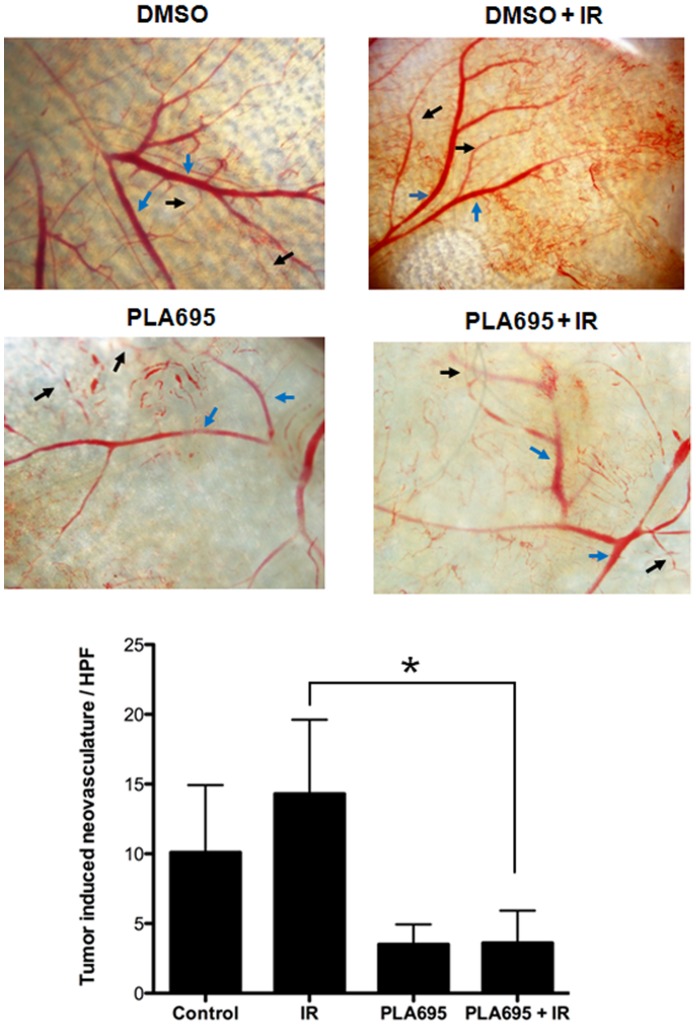
Combination treatment of PLA-695 with irradiation decreases vascularity. The C57/Bl6 mice were implanted with diffusion chambers containing LLC cells. Seven days after implantation, the mice were treated with PLA-695 (7.5 mg/Kg of body weight) or vehicle control for 30 min prior to IR (3 Gy). Fourteen days after implantation, the animals were sacrificed and the skin fold covering the diffusion chamber was analyzed. Shown are representative micrographs indication the tumor-induced neovasculature (black arrow) and pre-existing vasculature (blue arrow) and the bar graph depicting the mean number of neovasculature (>10 field for each mouse) SEM from each treatment group of 5 mice; * p<0.05.

We used a dorsal air sac model to study the effects of PLA-695 on tumor angiogenesis *in- vivo.* A chamber containing LLC cells was inserted in the dorsal skin fold and treated with DMSO (vehicle), IR alone (3 Gy), PLA-695 alone (7.5 mg/kg/day, intraperitoneal), or combination of PLA-695 and IR. Mice treated with IR showed increased microvasculature formation compared to the controls. However, mice treated with PLA-695 alone or in combination with IR showed destruction of microvasculature (Fig. 9). PLA-695 treated LLC tumors showed a significant decrease in microvasculature within the window model as compared to untreated control or radiation alone (p<0.05).

## Discussion

Previous studies have shown that cPLA_2_ is associated with tumor progression and radioresistance of the tumor vasculature [16], [17], [26]. cPLA_2_ is highly expressed in NSCLC [31], [32]. Through the Washington University-Pfizer alliance, we obtained inhibitors of cPLA_2_ with properties that are suitable for clinical trials. PLA-695 is a potent and selective cPLA_2_ inhibitor that is orally bio-available and has been the subject of Phase I and II clinical trials aimed to determine its analgesic effect in sufferers of chronic osteoarthritis (clinical trials.gov). Data from the clinical trials showed that PLA-695 was effective analgesic when compared to naproxen. Here we show that PLA-695 inhibited radiation-induced signal transduction. Specifically, treatment with PLA-695 led to a dose-dependent attenuation of radiation-induced ERK phosphorylation in HUVEC cells (Fig. 1). The Akt phosphorylation that occurs minutes following exposure to IR was also reduced to baseline levels in these cells. Similar phosphorylation of ERK and Akt was also seen in 3B11 cells. Clonogenic assays indicated that inhibition of cPLA_2_ with PLA-695 did not sensitize LLC or A549 cells to radiation (Fig. 2A). However, when the cancer cells (LLC and A549) were grown in co-cultures with endothelial cells (bEND3 and HUVEC) to simulate to tumor microenvironment, and then treated with PLA-695, the cancer cells were radiosensitized (Fig. 2B). These results indicated that the endothelium in the tumor microenvironment may play an important role in the response of the tumor to irradiation.

Treatment of endothelial and lung cancer cells with PLA-695 reduced proliferation at all the time points tested. On the other hand, the combination of PLA-695 with IR led to further reduction in proliferation at 48 h, 72 and 96 h (Fig. 3A). Treatment with the combination of PLA-695 and IR in LLC and A549 cells led to a treatment-time dependent increase of cell death, as monitored by annexin V and PI (Fig. 3B). We have shown earlier that cell death in endothelial cells after treatment with the cPLA2 inhibitor AACOCF_3_ was associated with mitotic catastrophe [17]. PLA-695 induced cell death increased at 96 hours post irradiation is similar to what we had reported earlier; thus, the cell death induced by a combined treatment of PLA-695 and IR is likely to be due to mitotic catastrophe (Fig. 4).

Consideration of the data from the clonogenic, proliferation and cell death studies leads to the conclusion that the treatments with a combination of PLA-695 and IR led to increased cell death contributing to reduced proliferation. Although PLA-695 is able to reduce proliferation of both endothelial cells and tumor cells, radiosensitization of tumor cells, LLC &A549, was detected only when they were grown in co-culture and not as independent cultures. Hence PLA-695 was effective as a radiosensitizer in the tumor microenvironment. Invasion and migration are crucial for tumorigenesis and proliferation of tumor cells. We found that inhibition of cPLA_2_ by PLA-695 prior to IR significantly attenuated invasion of LLC and A549 cells (Fig. 6). Combination of PLA-695 and IR also reduced migration in HUVEC and bEND3 cells (Fig. 5). Our findings in the heterotopic NSCLC mouse tumor models add to the results of, previous studies, which showed decreased tumorigenesis in mice genetically deficient in cPLA_2_ or those treated with chemical inhibitors of the enzyme [16], [26]. Tumors grown in cPLA2−/− mice are smaller than those grown in wild type mice [26]. In addition, tumor models treated with a lipid-mimetic inhibitor of PLA2 exhibited a significant tumor growth and decreased vascularity and perfusion [16]. In the current study we found that PLA-695 exhibits a similar effect in animal models of NSCLC. We have shown that combination therapy with IR results in a statistically significant tumor growth delay in both LLC and A549 lung tumor models (Fig. 7). Most significantly, A549 tumors failed to grow in 50% of the mice treated with PLA-695 and IR. The mechanism of action of PLA-695 also concurs with previous studies, as combination treated LLC tumors possessed significantly less vascular cross-sectional area and significantly suppressed the formation of microvessels and vascular network (Fig. 8). Results from the tumor vascular window model (Fig. 9) also indicated that PLA-695 is anti-angiogenic when administered alone or in combination with IR. Therefore, we have shown that cPLA_2_ plays an important role in tumor blood vessel formation. Thus cPLA2 is an attractive novel target for lung cancer treatment.

The radioresistance of tumor vasculature to conventionally fractionated radiotherapy in difficult to treat cancers is well documented [33]. While anti-angiogenic therapy is most certainly not a new idea, with multiple agents having been developed in an effort to inhibit tumor growth, these agents are rarely combined with radiation and, by themselves, usually result only in transitory clinical improvements that are subsequently followed by increased drug resistance [34], [35], [36]. Thus, the development of novel and effective drugs that can radiosensitize the tumor vasculature without serious side effects should improve the efficacy of tumor control by radiation. In this study, we identified and characterized PLA-695 as a selective and potent cPLA_2_ inhibitor capable of radiosensitizing vascular endothelial cells and animal models of lung cancer. Given PLA-695’s favorable pharmacological properties, combined with the results from this study, clinical testing of this agent in NSCLC is warranted.
